# A238 ALTERED GUT MICROBIOME COMPOSITION AND FUNCTION ARE ASSOCIATED WITH GUT BARRIER DYSFUNCTION IN HEALTHY RELATIVES OF CROHN’S DISEASE PATIENTS

**DOI:** 10.1093/jcag/gwab049.237

**Published:** 2022-02-21

**Authors:** H Leibovitzh, S Lee, M Xue, J Raygoza Garay, C Hernandez-Rocha, K Madsen, J Meddings, D S Guttmen, O Espin Garcia, A Goethel, A Griffiths, P Moayyedi, H Q Huynh, K Jacobson, D R Mack, M Abreu, C N Bernstein, J Marshall, D Turner, W Xu, W Turpin, K Croitoru

**Affiliations:** 1 University of Alberta, Edmonton, AB, Canada; 2 University of Miami School of Medicine, Miami, FL; 3 McMaster University Medical Centre, Hamilton, ON, Canada; 4 Shaare Zedek Medical Center, Jerusalem, Jerusalem, Israel; 5 University of Toronto Dalla Lana School of Public Health, Toronto, ON, Canada; 6 University of Toronto Temerty Faculty of Medicine, Toronto, ON, Canada; 7 Zane Cohen Centre for Digestive Diseases, Mount Sinai Hospital, Toronto, ON, Canada; 8 University of Calgary Cumming School of Medicine, Calgary, AB, Canada; 9 University of Toronto Department of Cell and Systems Biology, Toronto, ON, Canada; 10 The Hospital for Sick Children, Toronto, ON, Canada; 11 McMaster University Department of Medicine, Hamilton, ON, Canada; 12 BC Children’s Hospital, Vancouver, BC, Canada; 13 University of Ottawa, Ottawa, ON, Canada; 14 University of Manitoba, Winnipeg, MB, Canada

## Abstract

**Background:**

The gut microbiome may play a role in gut barrier homeostasis including epithelial barrier function, but data are scarce and limited to animal studies

**Aims:**

To assess if alterations in gut microbiome are associated with gut barrier function

**Methods:**

We utilized the Genetic Environmental Microbial (CCC GEM) cohort of healthy first-degree relatives (FDRs) of Crohn’s disease (CD) patients. Gut barrier function was assessed using the ratio of urinary fractional excretion of lactulose to mannitol (LMR). Stool bacterial DNA was extracted and sequenced for the V4 hypervariable region of the 16S rRNA gene using MiSeq and processed using QIIME2. Microbial functions were imputed using PICRUSt2. The cohort was divided into a North American discovery cohort (n=2,472) and non-North American external validation cohort (n=655). LMR>0.025 was defined as abnormal. LMR-microbiome associations were assessed using multivariable regression model and Random Forest (RF) classifier algorithm. q<0.05 was considered significant when multiple tests were performed

**Results:**

The median age of the entire cohort was 17.0 years [IQR 12.0; 24.0], 52.6% were females and 25.4% had LMR>0.025. In the discovery cohort, subjects with LMR>0.025 had markedly reduced alpha diversity (Chao1 index, estimate= -0.0037, p=4.0e-04) and altered beta diversity (Bray-Curtis dissimilarity index, PERMANOVA: pseudo-F statistic = 2.99, p=1.0e-03). We identified eight bacterial genera and 52 microbial pathways associated with LMR>0.025 (q<0.05). Four genera (decreased *Adlercreutzia* [odds ratio(OR)=0.74, 95% confidence interval (CI) 0.6–0.91], *Clostridia-UCG-014* [OR=0.71, 95%CI 0.59–0.86], and *Clostridium-sensu-stricto-1* [OR=0.75, 95%CI 0.61–0.92] and increased *Colidextribacter* [OR=1.65, 95%CI 1.2–2.26]) and eight pathways (including decreased biosynthesis of glutamate [OR=0.4, 95%CI 0.21–0.74], tryptophan [OR=0.06, 95%CI 0.01–0.27] and threonine [OR=0.038, 95%CI 0.003–0.41]) were replicated. Bacterial community composition was associated with gut barrier homeostasis as defined by the RF analysis (p= 1.4e-6)

**Conclusions:**

Gut microbiome community and pathways are associated with gut barrier function. These findings may identify potential microbial targets to modulate barrier function

Submitted on behalf of the CCC-GEM Consortium

**Funding Agencies:**

CCC, CIHRCrohn’s and Colitis Canada Genetics Environment Microbial (CCC-GEM) III; The Leona M. and Harry B. Helmsley Charitable Trust; Kenneth Croitoru is the recipient of the Canada Research Chair in Inflammatory Bowel Diseases

